# Supplementary material for: High Stability and Low Irritation of Retinol Propionate and Hydroxypinacolone Retinoate Supramolecular Nanoparticles with Effective Anti-Wrinkle Efficacy
Source: Pharmaceutics. 2023 Feb 22;15(3):731. doi: 10.3390/pharmaceutics15030731 (PMC10051651; doi:10.3390/pharmaceutics15030731)
Supplement: Supplementary file 1 [file pharmaceutics-15-00731-s001.zip › pharmaceutics-2162273-supplementary.pdf]

High stability and low irritation of Retinol Propionate and Hydroxypinacolone Retinoate Supramolecular Nanoparticles with effective Anti-wrinkle Efficacy

De Bai [1,†], Fan Hu [3,4,†], Huixian Xu [2], Jiahong Huang [1], Chengyu Wu [1,2,*], Jiaheng Zhang [1,2,*] and Rui Ye [3,4,*]

[1] Sauvage Laboratory for Smart Materials, Harbin Institute of Technology, Shenzhen 518055, China; 20b955043@stu.hit.edu.cn (D.B.); huangjiahong@stu.hit.edu.cn (J.H.);
[2] Shenzhen Shinesky Biological Technology Co., Ltd., Shenzhen 518055, China; xuhuixian@shinesky.com.cn (H.X.)
[3] Inertia Shanghai Biotechnology Co., Ltd., Shanghai, China
[4] DermaHealth Shanghai Biotechnology Co., Ltd., Shanghai, China

[†] These authors contributed equally to this work.

Corresponding authors:

Chengyu Wu: wuchengyu@hit.edu.cn,

Jiaheng Zhang: zhangjiaheng@hit.edu.cn,

Rui Ye: rye@uniskin.com.

Table 1. Physicochemical parameters.

	Viscosity (mPa·s)	Density (g/cm^3)	Conductivity (μs/cm)	pH	Appearance
Gravi-A nanoparticles	13.2	1.015	329	6.82	Yellowish Transparent Liquid

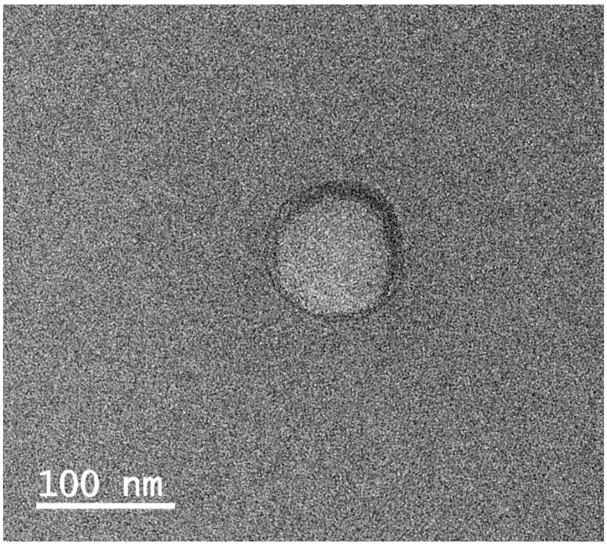

Figure S1. TEM image of the supramolecular Gravi-A nanoparticles (Scale bar 100nm)

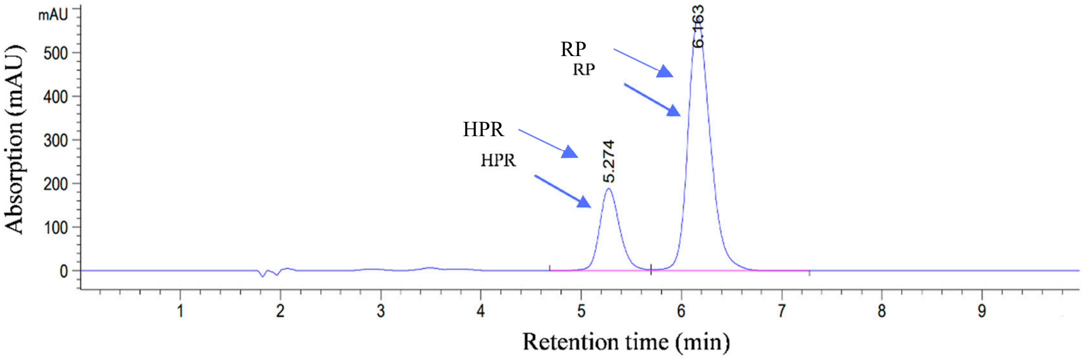

Figure S2. Peak spectra of Gravi-A nonoparticles detected in HPLC

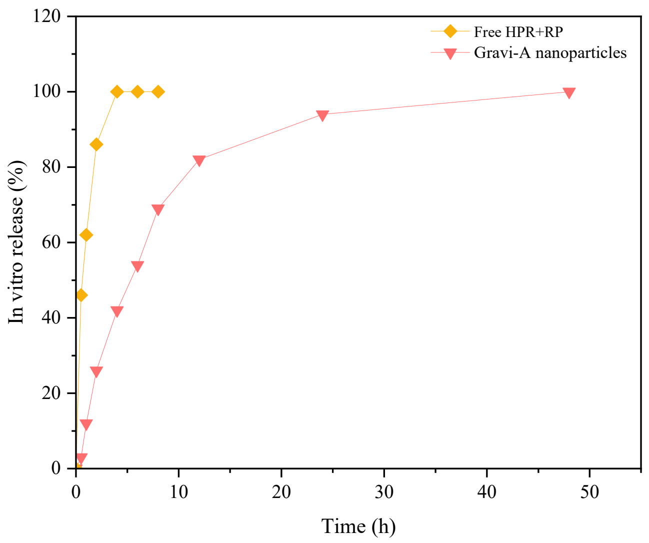

Figure S3. In vitro release curve

Table S2. Results of toxic effect of samples on chorioallantoic membrane of chick embryo.

**Sample**	**Concentration (%)**	**Number of chicken embryos**	**Number of positive reactions**	**RC_{50}[a]**
Gravi-A nanparticles	50	3	0	94.61% (95% NaCl: 71.96%–271.29%)
	30	10	0	
	50		2	
	80		4	
	100		5	
Free HPR + RP	50	3	0	＞ 100% (95% NaCl: N.D.)
	80	10	2	
	100		3	

[a]The results showed no irritation (RC_{50} ＞ 3.0 %), unpredictable (RC_{50} = 1.0 %–3.0 %), irritant (RC_{50} ＜ 1.0 %).

Table S3. Results of skin irritation score of supramolecular bis- A nanoparticles in New Zealand rabbits[a]

**Number**	**Skin irritation response**	**Observation days**													
		**1**	**2**	**3**	**4**	**5**	**6**	**7**	**8**	**9**	**10**	**11**	**12**	**13**	**14**
1	Erythema	0	0	0	0	1	1	1	1	1	1	1	1	1	1
	Edema	0	0	0	0	0	0	0	0	0	0	0	0	0	0
2	Erythema	0	0	0	0	0	1	1	1	1	1	1	1	1	1
	Edema	0	0	0	0	0	0	0	0	0	0	0	0	0	0
3	Erythema	0	0	0	0	1	1	1	1	1	1	1	1	1	1
	Edema	0	0	0	0	0	0	0	0	0	0	0	0	0	0
4	Erythema	0	0	0	0	0	1	1	1	1	1	1	1	1	1
	Edema	0	0	0	0	0	0	0	0	0	0	0	0	0	0
Average points per animal per day $= \frac{\sum \text{Total integral of erythema and edema}}{\text{Number of animals tested}} \div 14 =$													0.68		

The results were recorded according to the skin reaction grading standard in 6.4 of Cosmetic Technical Specifications (2015 edition). Erythema formation: no erythema (0), mild erythema (1), obvious erythema (2), moderate to severe erythema (3), severe erythema to mild eschar formation (4). Edema formation: no edema (0), mild edema (1), mild edema (2), moderate edema (3), severe edema (4). The results showed no irritation (0–0.5), light irritation (0.5–2.0), moderate irritation (2.0–6.0) and strong irritation (6.0–8.0).

Table S4. Results of skin irritation score of free HPR + RP in New Zealand rabbits[a]

Number	Skin irritation response	1	2	3	4	5	6	7	8	9	10	11	12	13	14
		**Observation days**													
1	Erythema	0	0	1	1	1	1	1	1	1	1	1	1	1	1
	Edema	0	0	0	0	0	0	0	0	0	0	0	0	0	0
2	Erythema	0	0	0	1	1	1	1	1	1	1	1	1	1	1
	Edema	0	0	0	0	0	0	0	0	0	0	0	0	0	0
3	Erythema	0	0	1	1	1	1	1	1	1	1	1	1	1	1
	Edema	0	0	0	0	0	0	0	0	0	0	0	0	0	0
4	Erythema	0	0	1	1	1	1	1	1	1	1	1	1	1	1
	Edema	0	0	0	0	0	0	0	0	0	0	0	0	0	0

Average points per animal per day $= \frac{\sum \text{Total integral of erythema and edema}}{\text{Number of animals tested}} \div 14 =$ 0.84

The results were recorded according to the skin reaction grading standard in 6.4 of Cosmetic Technical Specifications (2015 edition). Erythema formation: no erythema (0), mild erythema (1), obvious erythema (2), moderate to severe erythema (3), severe erythema to mild eschar formation (4). Edema formation: no edema (0), mild edema (1), mild edema (2), moderate edema (3), severe edema (4). The results showed no irritation (0–0.5), light irritation (0.5–2.0), moderate irritation (2.0–6.0) and strong irritation (6.0–8.0).

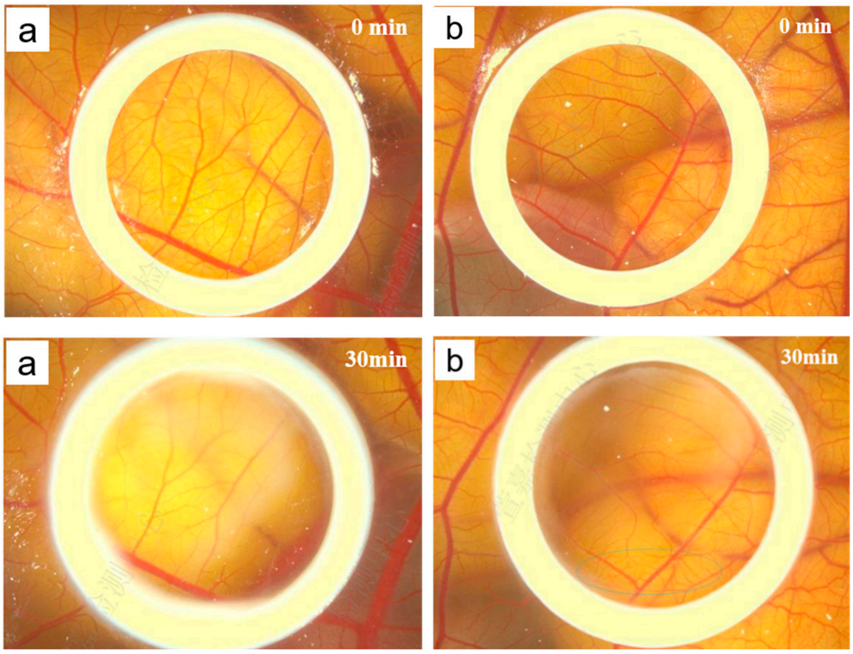

Figure S4. Effect of free HRP+PR on chorioallantoic membrane toxicity of chicken embryo after 30 min administration at different concentrations. 4% sample concentration in group a and 5% sample concentration in group b, where the yellow circle represents the selected viewing area.

Table S5. Summary of the results of patch test of bis- A nanoparticles on human skin[a]

Group	Number of subjects	Observation time (h)	Number of people with different skin reaction levels in patch test				
			0	1	2	3	4
5 % Gravi-A nanparticles	32	0.5	32	0	0	0	0
		24	32	0	0	0	0
		48	32	0	0	0	0
7 % Gravi-A nanparticles	32	0.5	32	0	0	0	0
		24	32	0	0	0	0
		48	32	0	0	0	0
10 % Gravi-A nanparticles	32	0.5	32	0	0	0	0
		24	32	0	0	0	0
		48	32	0	0	0	0
Negative control (blank + filter)	32	0.5	32	0	0	0	0
		24	32	0	0	0	0
		48	32	0	0	0	0

The results were recorded according to the skin reaction grading standard in 6.4 of Cosmetic Technical Specifications (2015 edition). Erythema formation: no erythema (0), mild erythema (1), obvious erythema (2), moderate to severe erythema (3), severe erythema to mild eschar formation (4).

0D	7D	14D	28D	56D

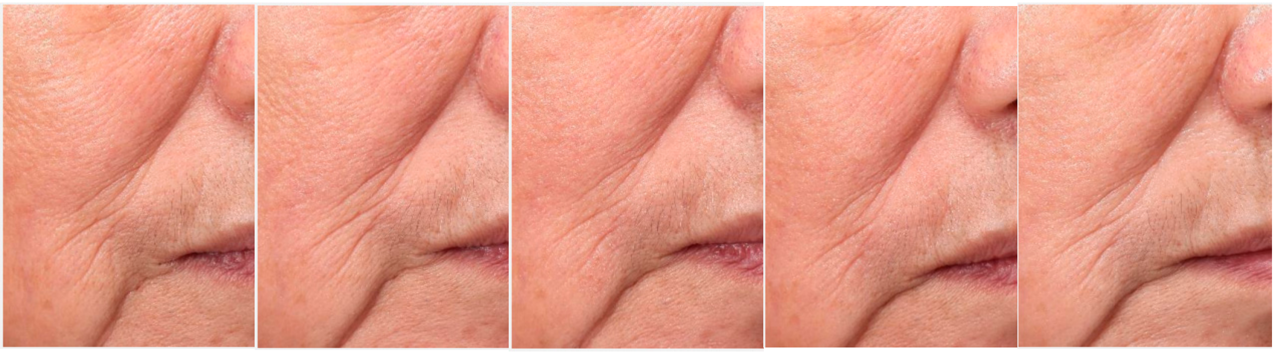

97.76 mm^2	86.39 mm^2	80.47 mm^2	65.98 mm^2	70.81 mm^2

Figure S5. Changes of nasolabial folds area in volunteer after using Gravi-A nanoparticles for 56 days

0D	7D	14D	28D	56D

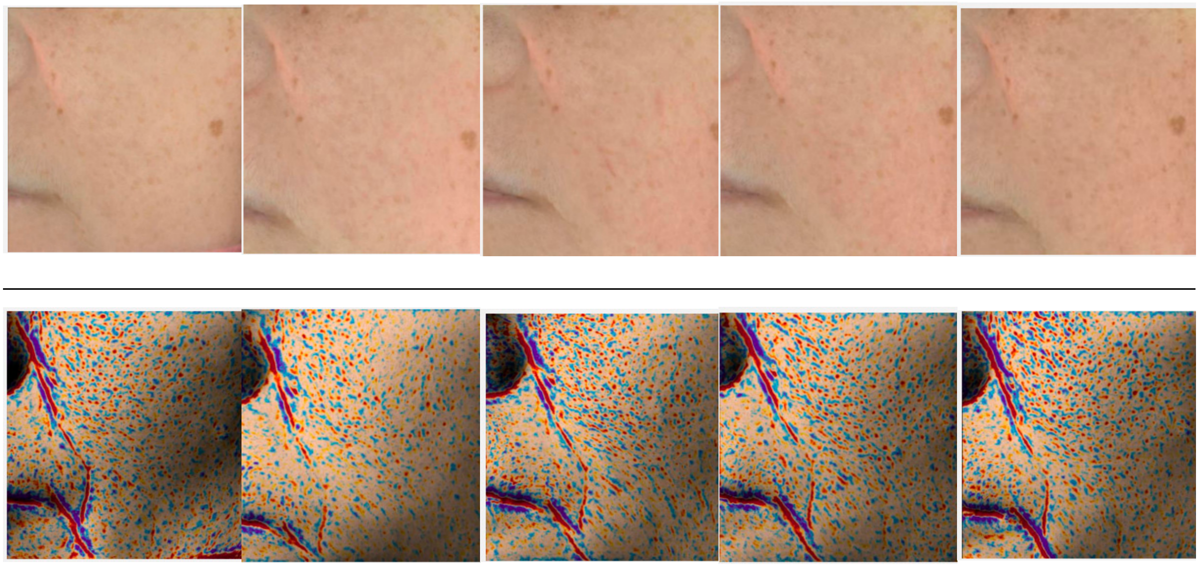

0.055 mm	0.027 mm	0.0323 mm	0.0317 mm	0.037 mm

Figure S6. Changes in the average depth of the volunteers' nasolabial folds after using Gravi-A nanoparticles for 56 days. The blue lines reflect the smoothness and fullness of the skin, and the purple lines reflect the smoothness and fullness of the skin.

0D	7D	14D	28D	56D

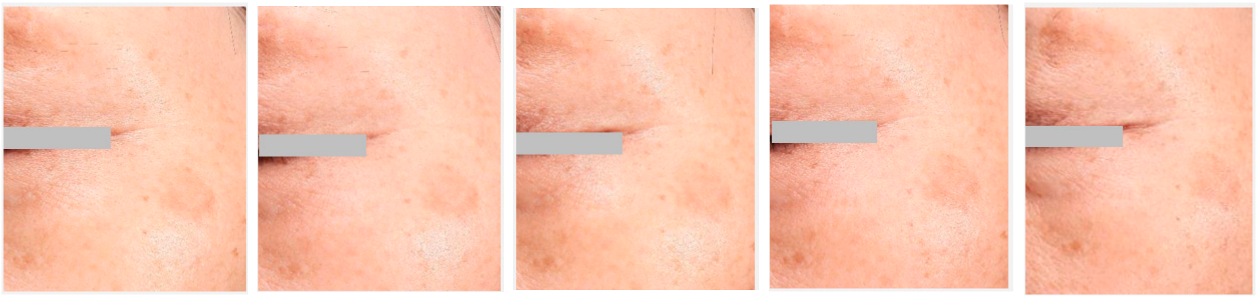

77.14 mm^2	56.53 mm^2	52.38 mm^2	52.65 mm^2	53.11 mm^2

Figure S7. Changes of crow's feet area in volunteer after using Gravi-A nanoparticles for 56 days

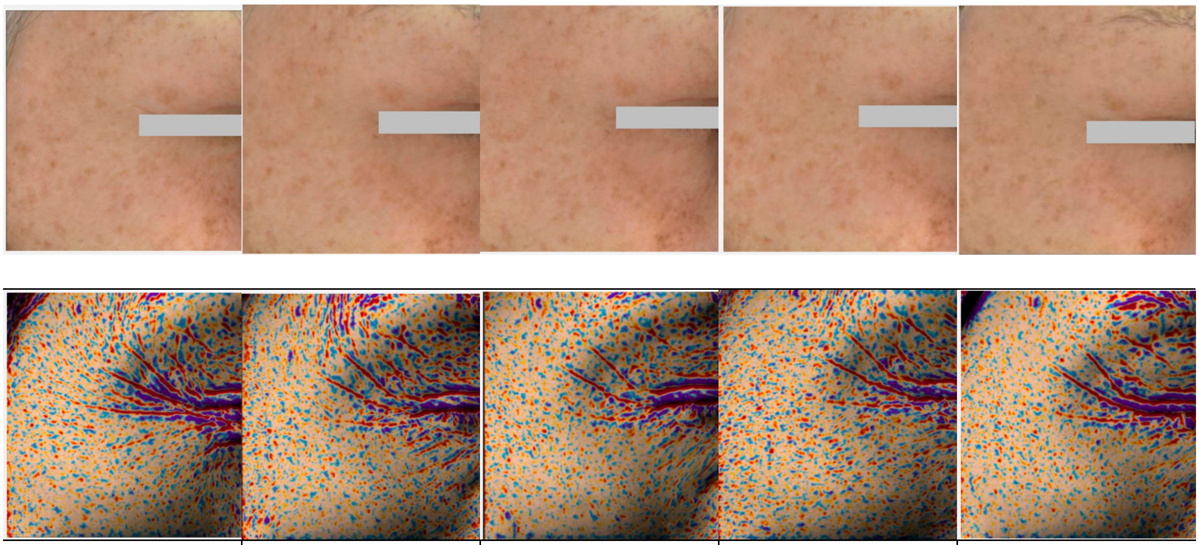

0D	7D	14D	28D	56D
24.67 mm	15.32 mm	13.52 mm	16.29 mm	15.88 mm

Figure S8. Changes in the length of crow's feet on volunteers' faces after using Gravi-A nanoparticles for 56 days. The blue lines reflect the smoothness and fullness of the skin, and the purple lines reflect the smoothness and fullness of the skin.

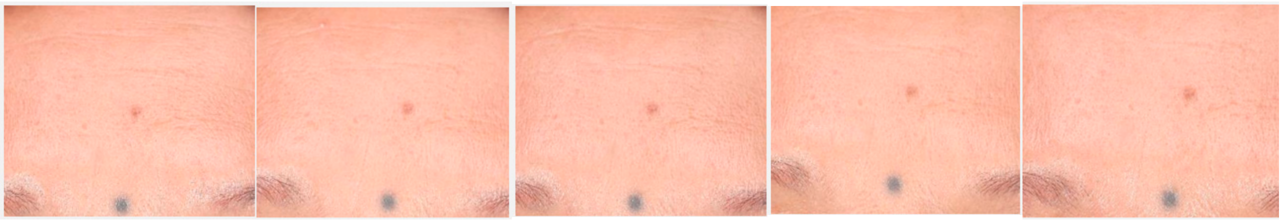

0D	7D	14D	28D	56D
205.45 mm^2	101.94 mm^2	136.22 mm^2	67.21 mm^2	89.83 mm^2

Figure S9. Changes of forehead wrinkles area in volunteer after using Gravi-A nanoparticles for 56 days

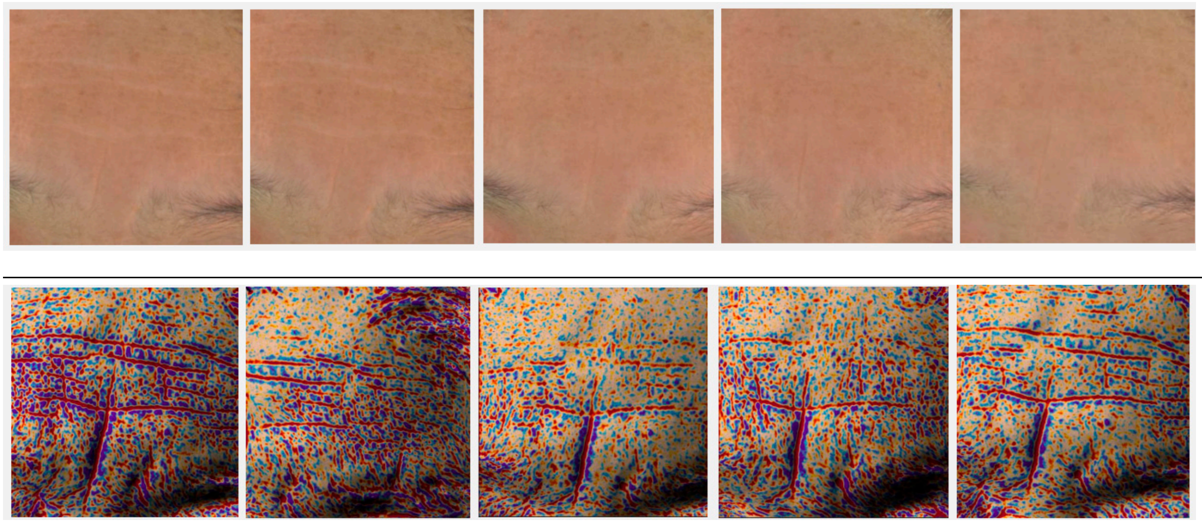

0D	7D	14D	28D	56D
0.038 mm	0.033 mm	0.023 mm	0.028 mm	0.037 mm

Figure S10. Changes in the average depth of the volunteers' forehead wrinkles after using Gravi-A nanoparticles for 56 days. The blue lines reflect the smoothness and fullness of the skin, and the purple lines reflect the smoothness and fullness of the skin.

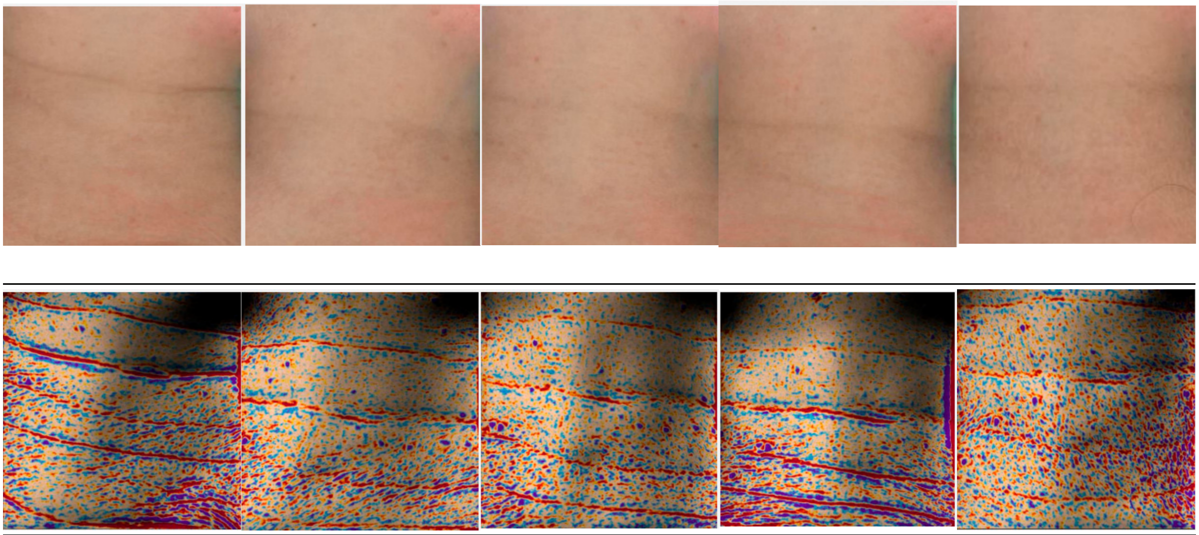

0D	7D	14D	28D	56D
0.90 mm	0.66 mm	0.77 mm	0.67 mm	0.71 mm

Figure S11. Changes in the average width of the volunteers' cervical stripe after using Gravi-A nanoparticles for 56 days. The blue lines reflect the smoothness and fullness of the skin, and the purple lines reflect the smoothness and fullness of the skin.

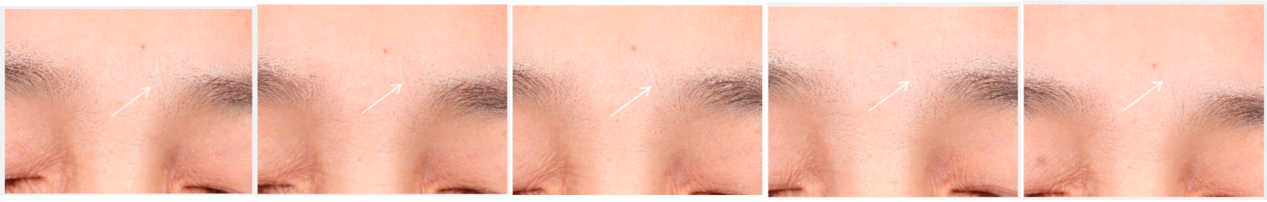

0D	7D	14D	28D	56D
83.74 mm^2	69.50 mm^2	67.59 mm^2	67.63 mm^2	66.01 mm^2

Figure S12. Changes of frown lines area in volunteer after using Gravi-A nanoparticles for 56 days, where the white arrows indicate areas that have changed significantly.

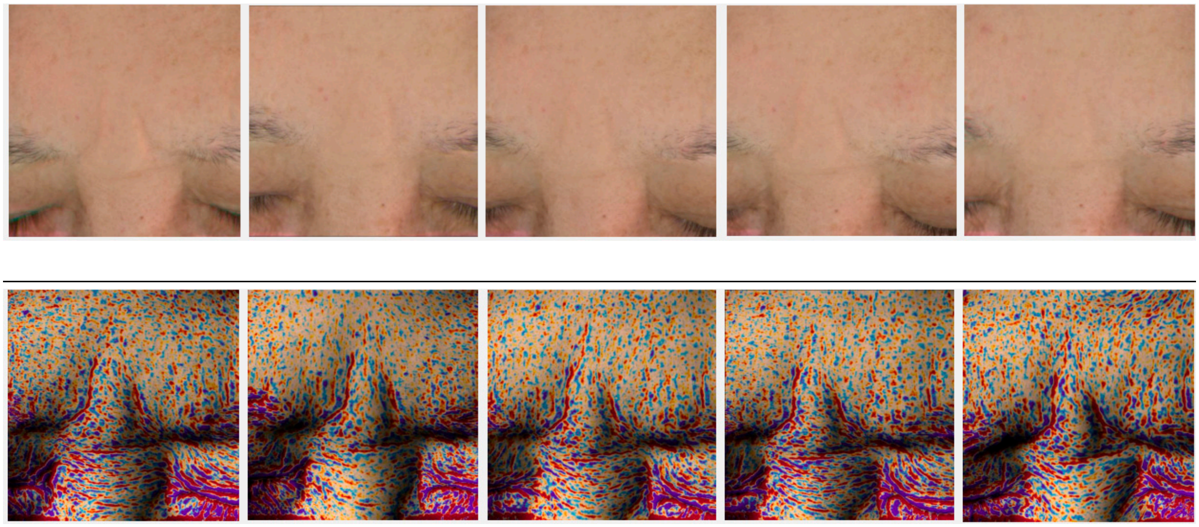

0D	7D	14D	28D	56D
0.90 mm	0.66 mm	0.77 mm	0.67 mm	0.71 mm

Figure S13. Changes in the length of the volunteers' frown lines after using Gravi-A nanoparticles for 56 days. The blue lines reflect the smoothness and fullness of the skin, and the purple lines reflect the smoothness and fullness of the skin.

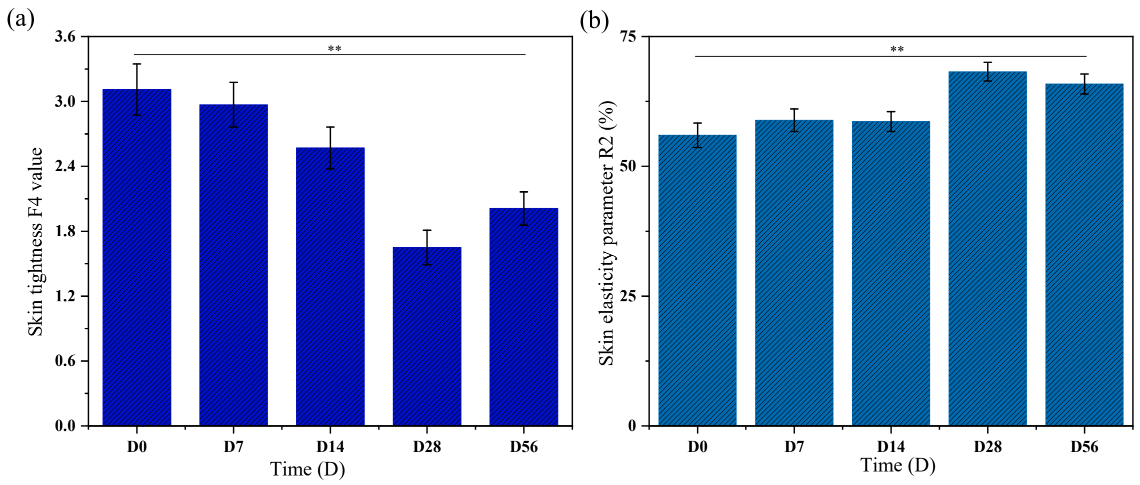

Figure S14. The degree of skin improvement within 56 days after using Gravi-A nanoparticles. (a) Detection value of skin tightness F4 at different time points. (b) Detection value of skin elasticity parameter R2 at different time points, ** $p < 0.01$.

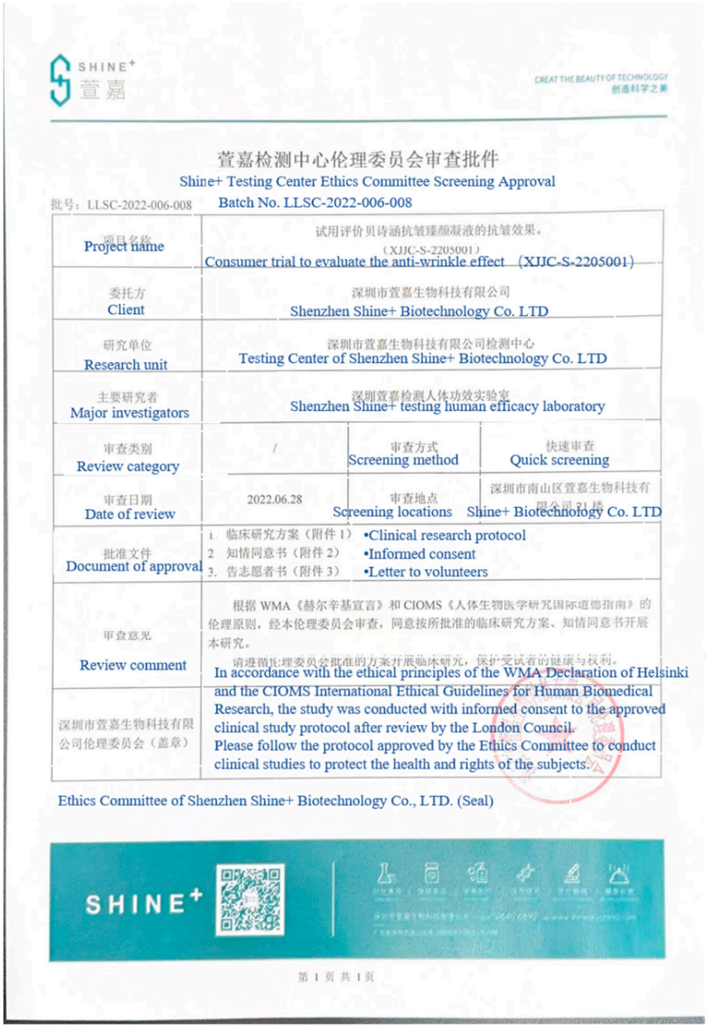

SHINE+ 萱嘉

CREAT THE BEAUTY OF TECHNOLOGY 创造科学之美

萱嘉检测中心伦理委员会审查批件

Shine+ Testing Center Ethics Committee Screening Approval

批号：LLSC-2022-006-008 Batch No. LLSC-2022-006-008

项目名称 Project name	试用评价贝诗涵抗皱臻颜凝液的抗皱效果。（XJJC-S-2205001） Consumer trial to evaluate the anti-wrinkle effect （XJJC-S-2205001）		
委托方 Client	深圳市萱嘉生物科技有限公司 Shenzhen Shine+ Biotechnology Co. LTD		
研究单位 Research unit	深圳市萱嘉生物科技有限公司检测中心 Testing Center of Shenzhen Shine+ Biotechnology Co. LTD		
主要研究者 Major investigators	深圳萱嘉检测人体功效实验室 Shenzhen Shine+ testing human efficacy laboratory		
审查类别 Review category	/	审查方式 Screening method	快速审查 Quick screening
审查日期 Date of review	2022.06.28	审查地点 Screening locations	深圳市南山区萱嘉生物科技有限公司21楼 Shine+ Biotechnology Co. LTD
批准文件 Document of approval	1. 临床研究方案（附件 1） •Clinical research protocol<br>2. 知情同意书（附件 2） •Informed consent<br>3. 告志愿者书（附件 3） •Letter to volunteers		
审查意见 Review comment	根据 WMA《赫尔辛基宣言》和 CIOMS《人体生物医学研究国际道德指南》的伦理原则，经本伦理委员会审查，同意按所批准的临床研究方案、知情同意书开展本研究。<br>请遵循伦理委员会批准的方案开展临床研究，保护受试者的健康与权利。<br>In accordance with the ethical principles of the WMA Declaration of Helsinki and the CIOMS International Ethical Guidelines for Human Biomedical Research, the study was conducted with informed consent to the approved clinical study protocol after review by the London Council.<br>Please follow the protocol approved by the Ethics Committee to conduct clinical studies to protect the health and rights of the subjects.		
深圳市萱嘉生物科技有限公司伦理委员会（盖章）			

Ethics Committee of Shenzhen Shine+ Biotechnology Co., LTD. (Seal)

SHINE+

第 1 页 共 1 页

Figure S15. Ethics Screening Document
